# Recent Advances in the Cellular and Molecular Mechanisms of Hypothalamic Neuronal Glucose Detection

**DOI:** 10.3389/fphys.2017.00875

**Published:** 2017-11-14

**Authors:** Xavier Fioramonti, Chloé Chrétien, Corinne Leloup, Luc Pénicaud

**Affiliations:** ^1^NutriNeuro, Institut National de la Recherche Agronomique, Université de Bordeaux, Bordeaux, France; ^2^Centre des Sciences du Goût et de l'Alimentation, Centre National de la Recherche Scientifique, Institut National de la Recherche Agronomique, Université Bourgogne Franche-Comté, Dijon, France; ^3^Stromalab, Centre National de la Recherche Scientifique, Institut National de la Santé et de la Recherche Médicale, Université de Toulouse, Toulouse, France

**Keywords:** glucose, hypothalamus, pro-opiomelanocortin neurons, transient receptor potential channels, reactive oxygen species, electrophysiology

## Abstract

The hypothalamus have been recognized for decades as one of the major brain centers for the control of energy homeostasis. This area contains specialized neurons able to detect changes in nutrients level. Among them, glucose-sensing neurons use glucose as a signaling molecule in addition to its fueling role. In this review we will describe the different sub-populations of glucose-sensing neurons present in the hypothalamus and highlight their nature in terms of neurotransmitter/neuropeptide expression. This review will particularly discuss whether pro-opiomelanocortin (POMC) neurons from the arcuate nucleus are directly glucose-sensing. In addition, recent observations in glucose-sensing suggest a subtle system with different mechanisms involved in the detection of changes in glucose level and their involvement in specific physiological functions. Several data point out the critical role of reactive oxygen species (ROS) and mitochondria dynamics in the detection of increased glucose. This review will also highlight that ATP-dependent potassium (K_ATP_) channels are not the only channels mediating glucose-sensing and discuss the new role of transient receptor potential canonical channels (TRPC). We will discuss the recent advances in the determination of glucose-sensing machinery and propose potential line of research needed to further understand the regulation of brain glucose detection.

Glucose homeostasis needs to be highly regulated to maintain blood glucose level constant. This is mandatory avoid any drop in blood glucose level as this nutrient is the preferred fuel source for the brain. Furthermore, increased blood glucose level also needs to be controlled to prevent long-term complication of excessive energy supply. Detection of changes in glucose level is achieved by glucose-sensors which work in concert to maintain glucose homeostasis. They are located at several anatomical sites at the periphery including taste buds of the tongue, the carotid bodies, the intestine, the portal vein, and the endocrine pancreas. In the central nervous system these sensors, called glucose-sensing neurons, are also localized in different brain areas. These neurons have initially been characterized in the hypothalamus. Even if they seem enriched within different hypothalamic nuclei, they have also been found in other areas including the brainstem, the cortex, the hippocampus, the thalamus, the amygdala, or the olfactory bulbs (see for review Steinbusch et al., 2015. Thus, glucose-sensing neurons have been suggested to be involved (1) in the control of feeding initiation and termination and (2) through the modulation of the autonomic nervous system, in functions modulated in response to changes in brain glucose levels among which pancreatic hormonal secretion, β-cell proliferation, thermogenesis, and hepatic glucose production. The presence of these neurons in extra-hypothalamic or brainstem structures suggests that they could be involved in other physiological functions than the control of energy homeostasis such as memory, olfaction, motivation, or reward for instance. However, characterization of physiological functions modulated by glucose-sensing needs further attention. In this review, we will discuss the recent advances made on the role of hypothalamic glucose-sensing neurons in the control of energy homeostasis.

## Characterization of hypothalamic glucose-sensing neurons

The idea that specialized cells could detect changes in glucose level originated from Oomura' and Anand's groups (Anand et al., 1964; Oomura et al., 1964). Later, Oomura and colleagues conclusively demonstrated the presence of specialized glucose-sensing neurons in showing that the direct electro-osmotic application of glucose altered the activity of hypothalamic neurons (Oomura et al., 1974). These neurons are now defined as cells able to adapt their electrical activity by directly detecting changes in extracellular glucose level. By definition, glucose-excited (GE) neurons increase their electrical activity when glucose level rises. By opposition, glucose-inhibited (GI) neurons increase their activity when glucose level decreases. It is important to note that glucose-sensing neurons directly detect changes in glucose level. In the brain, many neurons can see their electrical activity modulated by glucose but essentially due to pre-synaptic inputs coming from “true glucose-sensing” cells. Thus, in this review, we will refer as glucose-sensing cells, neurons which directly detect changes in glucose level.

**Figure 1:**
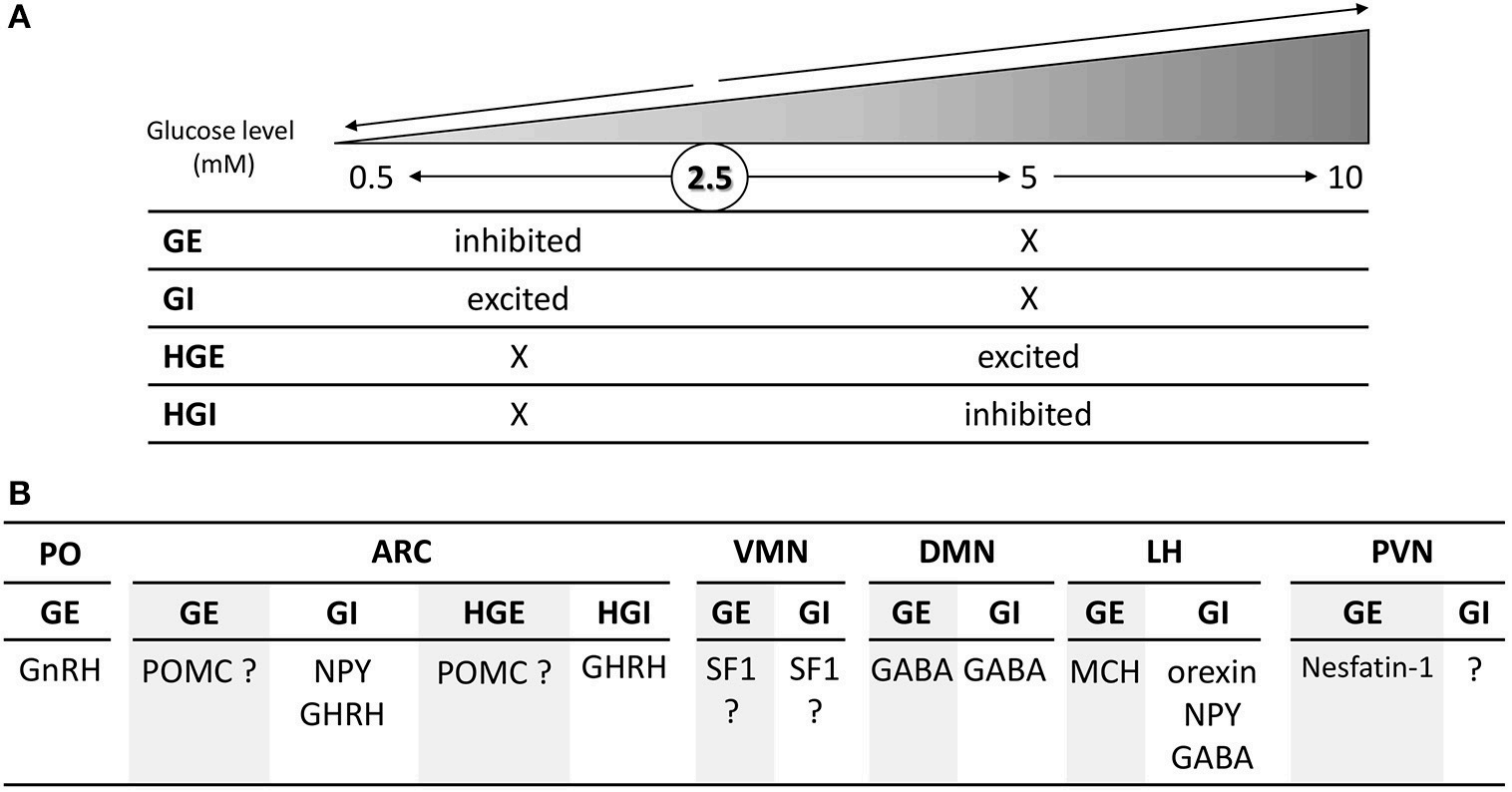
Schematic overview of the changes in glucose level detected **(A)** and nature **(B)** of the four subtypes glucose-sensing neurons present in the hypothalamus. **(A)** GE and GI neurons are respectively inhibited and activated by changes in glucose level below 2.5 mM and are non-sensitive to change above 2.5 mM. By opposition, HGE and HGI neurons are respectively activated and inhibited by changes above 2.5 or 5 mM while their activity is not altered by changes below 2.5 mM. The symbol “X” means that the electrical activity is not changed in comparison to the basal activity at 2.5 mM. **(B)** Nature of hypothalamic glucose-sensing neurons. ARC, arcuate nucleus; DMN, dorsomedian nucleus; LH, lateral hypothalamus; PVN, paraventricular nucleus; PO, pre-optic area; VMN, ventromedian nucleus. The symbol “?” means that the nature of glucose-sensing neurons has yet to be determined.

Within the medio-basal hypothalamus, which contains both the arcuate (ARC) and ventromedian nuclei (VMN), four populations of glucose-sensing neurons have been characterized according to the changes in glucose level they detect. Evidence suggest that specialized glucose-sensing neurons detect changes in glucose level either above or below 2.5 mM (Fioramonti et al., 2004; Wang et al., 2004; Penicaud et al., 2006; Chretien et al., 2017; Figure 1A). These neurons are referred as GE or GI neurons (respectively inhibited or activated in response to decreased glucose level below 2.5 mM) and HGE or HGI neurons (for high-glucose-excited or -inhibited, respectively activated or inhibited by changes above 2.5 mM). Interestingly, the electrical activity of GE and GI neurons is only changed in response to glucose change below 2.5 mM and not altered by changes in glucose level above 2.5 mM (Fioramonti et al., 2004; Wang et al., 2004). Similarly, we found that HGE and HGI neurons only change their electrical activity in response to changes in glucose level above 2.5 mM but not below this level (Fioramonti et al., 2004). Finding these different sub-populations of glucose-sensing neurons enriched the debate on the glucose level present in the hypothalamus.

### Brain glucose levels

The level of brain glucose is a process finely regulated. Glucose transporter 1 (GLUT1) seems to be the primary transporter controlling brain glucose entry (Cardoso et al., 2010). The high affinity of this transporter for glucose justifies the level found in the brain which is about 30% of the blood level and close to 2 mM at baseline (~7–8 mM blood glucose). This is the case in the hypothalamus or other areas including the hippocampus and striatum for instance (McNay and Gold, 1999; McNay et al., 2001; de Vries et al., 2003; Dunn-Meynell et al., 2009; Langlet et al., 2013). Interestingly, hypothalamic glucose level does not seem to fluctuate significantly between meals (Dunn-Meynell et al., 2009) or even after fasting (Langlet et al., 2013). Langlet and colleagues even found that glucose level in the ARC is slightly increased after fasting due to an increased permeability of the blood-brain-barrier (BBB). Thus, only profound manipulations of blood glucose level would induce changes in hypothalamic levels including insulin-induced hypoglycemia or pathological hyperglycemia. Such conditions would make hypothalamic levels to fluctuate between 0.2 and 4.5 mM (see for review Routh et al., 2014). Nevertheless, the finding of specialized neurons able to detect changes above 2.5 or even 5 mM suggests that, in confined areas, glucose level could be increased closer to level found in the blood. This could be the case around fenestrated capillaries of the BBB present in the ventral part of the ARC (Ciofi, 2011; Langlet et al., 2013). In support of this hypothesis, HGE and HGI neurons have only been found in the ARC (Fioramonti et al., 2004) and not in the VMN (Song et al., 2001). In this later study, neurons activated by increased glucose level above 5 mM have been found in the VMN but due to pre-synaptic glutamate inputs and not though a direct detection (Song et al., 2001). Local changes of glucose level around fenestrated capillaries would not be detectable by the available techniques including the microdialysis which does not present sufficient spatial resolution to monitor changes in confined environments. Determining (1) whether HGE- and HGI-like neurons can be found in extra-hypothalamic circumventricular organs in which the BBB is fenestrated (e.g., area postrema of the hindbrain, the subfornical organ and the vascular organ of lamina terminalis); (2) the real concentration of glucose in these micro-environments, will help characterizing brain spots containing these specialized glucose-sensing neurons as well as their physiological functions.

## Nature of hypothalamic glucose-sensing neurons

In order to better appreciate the physiological role of glucose-sensing neurons in the hypothalamus, several groups have dedicated means to determine the nature of these cells. Thus, studies have essentially tried to determine glucose-sensing properties of known neuropeptide-expressing neurons of the hypothalamus (Figure 1B). In the lateral hypothalamus (LH), orexin-, neuropeptide Y (NPY)-, and GABA-expressing neurons present GI-like properties whereas melanin-concentrating-hormone (MCH) neurons are GE-type (Williams et al., 2001; Burdakov et al., 2005; Gonzalez et al., 2008; Karnani and Burdakov, 2011; Marston et al., 2011; Sheng et al., 2014). In the paraventricular nucleus (PVN), some GE neurons have been shown to be nesfatin-1-expressing cells (Sedbazar et al., 2014). The identity of the remaining glucose-sensing neurons in this nucleus has yet to be determined. In the dorsomedian nucleus (DMN), some GABAergic neurons are GE or GI neurons (Otgon-Uul et al., 2016). In the VMN, GE or GI neurons have been shown to express the transcription factor Steroidogenic factor 1 (SF1; Toda et al., 2016). This would suggest that VMN glucose-sensing neurons are glutamatergic since most of SF1 neurons express this neurotransmitter (McClellan et al., 2006; Tong et al., 2007). This hypothesis is supported by the study of Tong et al. showing that the counter-regulation to hypoglycemia is impaired in mice lacking glutamate release in VMN SF1 neurons. Nevertheless, VMN GE or GI neurons could express other neurotransmitter or neuropeptide since glutamate is not the only transmitter expressed in this nucleus or even in SF1 neurons (McClellan et al., 2006). In the anterior part of the hypothalamus, GnRH (gonadotrophin releasing hormone) neurons of the preoptic area (PO) are GE (Roland and Moenter, 2011a,b; Beall et al., 2012). Interestingly, only GnRH neurons of this area seem to be glucose-sensing (Roland and Moenter, 2011b). In the ARC, we and others show that the large majority of GI neurons express NPY (Muroya et al., 1999; Fioramonti et al., 2007; Murphy et al., 2009). Stanley et al. also showed that ARC growth-hormone-releasing hormone (GHRH)-expressing neurons present GI- or HGI-like properties as they detect changes in the 0.2–10 or 4–10 mM windows level (Stanley et al., 2013). The nature of GE and HGE neurons is to debate as several studies show different results regarding their identity being POMC-expressing cells or not.

### Are POMC neurons glucose-sensing cells?

**Figure 2:**
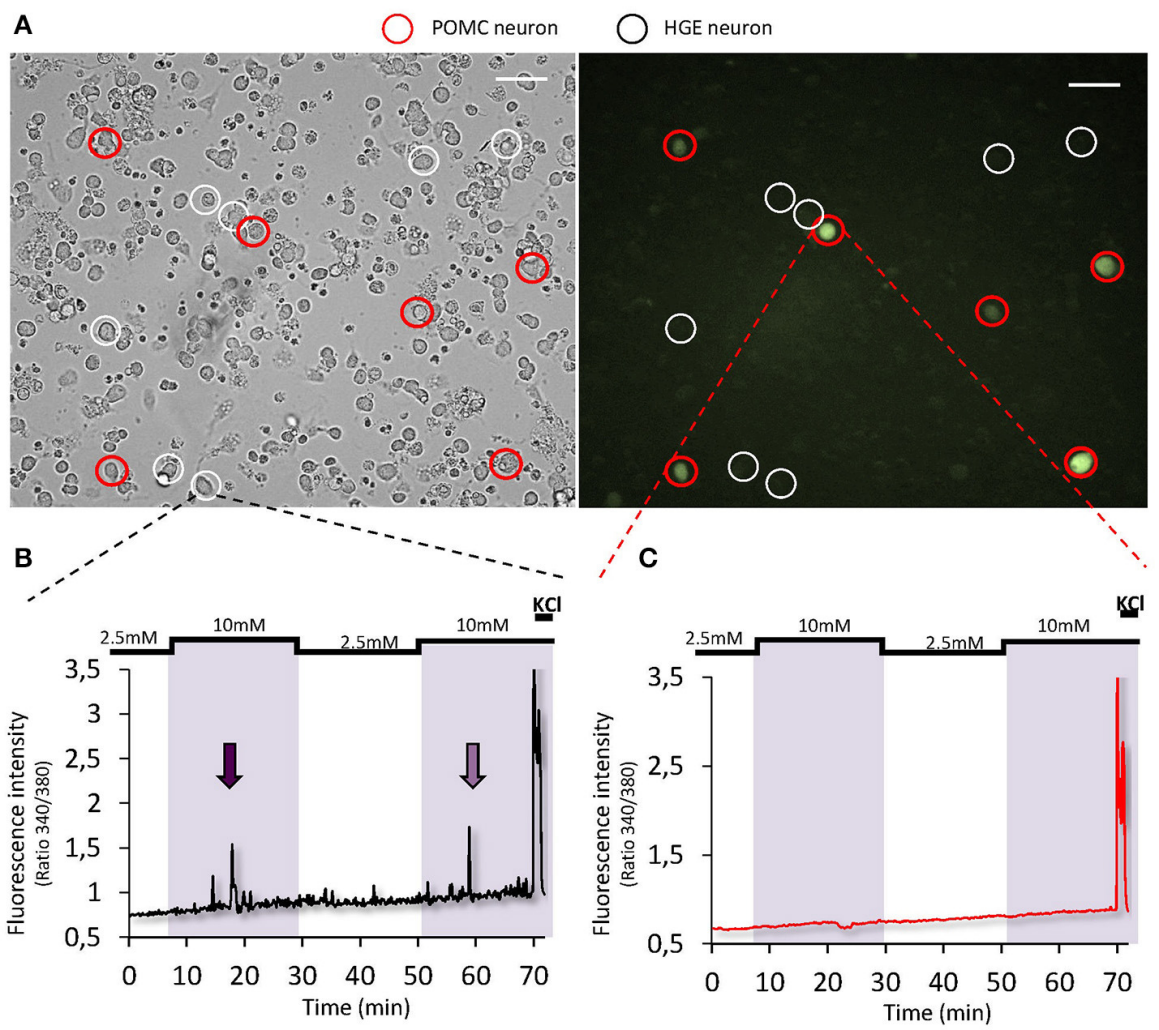
Glucose-sensing properties of POMC neurons. **(A)** Representative bright-field (left panel) or fluorescence (right panel) images of cultured dissociated MBH neurons from POMC-GFP mice (×20 objective, scale bar = 40 μm; Fioramonti et al., 2007). Detailed methods for cell culture preparation and calcium imaging recording is explained in Chretien et al. (2017). **(B,C)** Representative calcium imaging traces of a HGE **(B)** or a non-glucose-sensing POMC neuron **(C)** in response to 2 consecutives increased glucose level from 2.5 to 10 mM.

Some studies using electrophysiology on brain slices suggested that POMC neurons could be either GE or HGE-type neurons as their activity is modulated by changes in extracellular glucose level (Ibrahim et al., 2003; Claret et al., 2007; Parton et al., 2007). Other studies including one from our lab did not find POMC neurons directly glucose-sensing (Wang et al., 2004; Fioramonti et al., 2007). Nevertheless, a study from Parton et al. showed that α-melanocyte-stimulating hormone (α-MSH) release on hypothalamic chunks is increased in response to increased glucose level (Parton et al., 2007). This shows that POMC neurons can be activated by increased glucose level. Nevertheless, to our knowledge, no study has shown that glucose directly modulates the activity of POMC neurons. One could hypothesize that the modulation of POMC activity by glucose is due to presynaptic inputs rather than a direct detection by POMC neurons themselves. The hypothesis that POMC neurons would not detect changes in glucose level directly is supported by a recent work showing that the frequency of excitatory postsynaptic currents onto POMC neurons is modulated by glucose (Hu et al., 2014). To get further insight into the direct glucose-sensing properties of POMC neurons, we studied their activity using calcium-imaging on freshly dissociated hypothalamic cells from POMC-GFP mice (for detailed method, see Chretien et al., 2017). In such preparation, dissociated cells are not in contact from each other (Figure 2). Thus, this strategy allows the study of direct glucose-sensing properties of hypothalamic neurons (Dunn-Meynell et al., 2002; Kohno et al., 2003; Kang et al., 2004; Vazirani et al., 2013; Chretien et al., 2017). As presented in Figure 2, we found that ~8% hypothalamic neurons tested were identified as HGE neurons as they harbor a transient increase in [Ca^2+^]_i_ in response to a raise in glucose level from 2.5 to 10 mM. Nevertheless, none of the HGE neurons identified were GFP-expressing neurons (*n* = 11; Figure 2). Altogether, these data plus those from the literature suggest that glucose-sensing neurons modulate the melanocortin system but that POMC neurons themselves do not directly detect changes in glucose level. Further studies are still needed to determine the identity of ARC GE and HGE neurons. Knowing better the identity of hypothalamic (and extra-hypothalamic) glucose-sensing neurons is necessary to decipher the physiological functions controlled by these neurons.

## Molecular mechanisms involved in hypothalamic glucose-sensing neurons

The determination of the signaling pathways involved in the detection of changes in glucose level has aroused the scientific community for decades. Detailed mechanisms involved in GE and GI neurons can be found in the review from Routh et al. in this special issue. We will concentrate here on ionic channels and signaling pathways taking part in hypothalamic detection of increased glucose level. We will not discuss the role of the couple GLUT2/Glucokinase in the detection of increased glucose since it has been suggested a while ago. Interestingly, the most recent studies have rather shown their putative role in the detection of decreased glucose level and in the regulation of the counter-regulatory response to hypoglycemia (Dunn-Meynell et al., 2002; Marty et al., 2005; Kang et al., 2006).

### Which ion channel(s) are involved in glucose detection?

To determine mechanisms of brain glucose-sensing, studies have initially tried to determine similarities and differences from the known gold-standard glucose-sensor, the pancreatic β-cell (Yang et al., 1999). Thus, Michael Ashford was the first to show that ATP-dependent potassium (K_ATP_) channels are involved in glucose-sensing of GE neurons (Ashford et al., 1990a,b). Nevertheless, it is not possible to determine whether GE and/or HGE-like neurons were studied as those pioneer studies used large glucose changes from 0 to 10 mM. Later, studies from Vanessa Routh's laboratory demonstrated that K_ATP_ channels are indeed involved in the detection of decreased glucose level by GE neurons (2.5–0.1 mM level window; Song et al., 2001; Wang et al., 2004). Nevertheless, they showed that the change in K_ATP_ conductance plateau above 2.5 mM (Wang et al., 2004), suggesting that K_ATP_ channels could only mediate detection below this level, and consequently, only in GE neurons. In addition, Guy Rutter's group suggested that K_ATP_ channels would not be involved in glucose detection above 2.5 mM in a study showing that intracellular ATP level is not increased in hypothalamic neurons in response to increased glucose level from 3 to 15 mM (Ainscow et al., 2002). We confirmed that K_ATP_ channels are not involved in HGE neurons. Indeed, we found that these channels are essentially closed at 5 mM. We also showed that HGE neurons can be found in K_ATP_-deficient mice (Fioramonti et al., 2004) whereas GE neurons cannot be detected in these mice (Miki et al., 2001). Yang et al previously suggested that K_ATP_-independent mechanisms would be involved in 5–20 mM glucose detection as the K_ATP_-opener diazoxide failed to prevent increased glucose detection (Yang et al., 1999). Instead, we found that a non-selective cationic conductance was involved in the glucose response of HGE neurons (Fioramonti et al., 2004). More recently, we demonstrated that transient-receptor-potential canonical type 3 (TRPC3) channels are required for glucose detection by HGE neurons (Chretien et al., 2017). Involvement of non-selective cationic conductance has also been identified in the response to glucose of GnRH neurons even though the identity of the channel responsible for glucose-sensing in these neurons is yet to be determined (Roland and Moenter, 2011b). TRPC channels are involved in the response to insulin and leptin of ARC kisspeptin and POMC neurons (Qiu et al., 2010, 2011, 2014, 2017). Together, these studies open new line of research for hypothalamic glucose- and, more generally, nutrient-sensing mechanisms (Chretien et al., 2017).

### Role of the mitochondrial reactive oxygen species (mROS) in glucose detection

The mitochondrial respiratory chain represents one the main sources of ROS. Increasing reduced equivalents (NADH,H^+^ and FADH_2_) as a result of glucose oxidation leads to increased electron transfer chain activity, generating an elevated but physiological superoxide anions production. Our group showed that cerebral injection of glucose in rodents leads to a short-lived mROS signaling, in the form of H_2_O_2_ (see for review Leloup et al., 2011). We and others also showed that this increase in mROS level is necessary for the of glucose-sensing neurons to increased glucose (Chretien et al., 2017) and the regulation of food intake and insulin secretion (Leloup et al., 2006; Andrews et al., 2008; Colombani et al., 2009; Carneiro et al., 2012). Thus, increased mROS levels, rather than just the ATP/ADP ratio, constitute a signal that mediates the stimulatory effect of glucose on some hypothalamic neurons. Therefore, mROS signaling is consistent with the NADH mechanism suggested earlier for the glucose sensing mechanism (Yang et al., 1999; Ainscow and Rutter, 2002). In addition, the fact that TRPC3 channels form a redox-sensitive complex reinforces the role of mROS in the signaling involved in glucose detection.

Mitochondria are organized into a tubular network that continuously changes its shape and motility, mediated by fission and fusion mechanisms. Mitochondrial dynamics (fusion and fission) have been linked to the balance between energy demand and nutrient supply. Our group suggested that mitochondria dynamics (fission) play a significant role in mROS production in response to increased glucose level. In the MBH, we highlighted glucose-induced Drp1-dependent mitochondrial fission is an upstream regulator for mROS signaling, and consequently, a key mechanism in hypothalamic glucose sensing (Carneiro et al., 2012). More recently, Diano's and Claret's teams showed that fission and fusion mechanisms are involved in the detection of increased or decreased glucose level by hypothalamic POMC or SF1 neurons (Schneeberger et al., 2013; Toda et al., 2016; Ramirez et al., 2017; Santoro et al., 2017). Even though the involvement of mitochondria dynamics seems to be a critical mechanism involved in glucose detection and in the regulation of energy and glucose homeostasis, the signaling pathways leading to such changes in mitochondria morphology is unclear and needs to be studied further. The idea that such mechanisms are specific to glucose-sensing neurons also needs to be addressed.

### What about *metabolism-independent* glucose-sensing mechanisms?

Some studies highlighted that *metabolism-independent* mechanisms take part in neuronal glucose-sensing. Thus, it has been shown that the sodium-glucose cotransporters (SGLT)-inhibitor phloridzin, blocks the response to increased glucose level of some hypothalamic neurons (Yang et al., 1999; O'Malley et al., 2006). Furthermore, the non-metabolizable substrate of SGLTs, α-methylglucopyranoside, mimics the effect of increased glucose level on a majority of HGE-like neurons (O'Malley et al., 2006). SGLT1 or SGLT3 could be the transporters responsible for glucose detection as they are expressed in hypothalamic neurons. The hypothesis on SGLT3 is particularly interesting since this transporter is expressed in human cholinergic neurons where it acts as a glucose-activated sodium channel which does not transport glucose (Diez-Sampedro et al., 2003). Interestingly, SGLT3 has been shown to be the sensor mediating glucose detection of the portal vein (Delaere et al., 2012).

Another *metabolism-independent* mechanism has been suggested to mediate glucose detection in the hypothalamus through sweet taste receptors. These receptors present in papillae of the tongue mediate the sweet sensation of sugars and sugar substitute (Laffitte et al., 2014). These receptors are heterodimers composed of T1R2/T1R3 subunits which have also been identified in several extra-gustatory tissues and cells including the pancreatic β-cell and the brain (Laffitte et al., 2014). In the brain, the expression of T1R2 and T1R3 subunits is particularly enriched in the ARC (Ren et al., 2009; Herrera-Moro Chao et al., 2016). Recently, Yada's group showed that half of the HGE-like neurons in the ARC (activated by the increase in glucose level from 1 to 10 mM) are excited by the application of the sweetener sucralose. They also showed that the sweet taste receptor inhibitor gurmarin, blocks the response to glucose of 2/3 of HGE-like neurons (Kohno et al., 2016). Finally, in this study, they also show that sucralose activates neurons of the ARC which are not POMC neurons, reinforcing the idea that POMC neurons do not detect directly changes in glucose level. These data highlight a new mechanism involved in hypothalamic glucose-sensing. Further studies are however necessary to determine the physiological role of the sweet taste receptors expressed in hypothalamic glucose-sensing neurons in the control of energy homeostasis. It will also be interesting to determine the ionic channel mediating the effect of sweeteners or glucose downstream sweet taste receptor. The involvement of K_ATP_ or TRPC channels has always been linked to *metabolic-dependent* mechanisms. However, one could hypothesize that these channels mediate sweet taste receptor-dependent glucose-sensing. Twik-related acid-sensitive potassium-like (TASK) 1/3 channels could also be a relevant candidate as they take part in *metabolism-independent* mechanism of orexin GI neurons in the LH (Gonzalez et al., 2008, 2009).

## Conclusion

We described briefly the recent advances made in the comprehension of mechanisms involved in brain detection of increased glucose level. We highlighted the role of TRPC channels, ROS, mitochondria dynamics and pointed out that POMC neurons might not be directly sensing glucose. Nevertheless, additional studies are still needed to determine parts of the puzzle linking the pathways and whether they are activated in a same glucose-sensing neuron or whether several mechanisms are recruited in distinct neurons. We also highlighted that new lines of research are still needed to further characterize the nature of glucose-sensing neurons and to determine potential new locations within the brain. These data will help better understanding of the various physiological functions they control.

## Ethics statement

All procedures were performed in agreement with European Directive 2010/63/UE and approved by the French Ministry of Research and the local ethics committee of the University of Burgundy (C2EA Grand Campus Dijon N°105; agreement N°02404.02).

## Author contributions

Research data, CC. Wrote the manuscript, XF, LP, CC, and CL. Review/Edited the manuscript, XF, CC, LP, CL.

### Conflict of interest statement

The authors declare that the research was conducted in the absence of any commercial or financial relationships that could be construed as a potential conflict of interest. The reviewer GTD and handling Editor declared their shared affiliation.

## Abbreviations

α-MSH: α-melanocyte-stimulating hormone
ARC: arcuate nucleus
BBB: blood-brain-barrier
DMN: dorsomedian nucleus
GE neurons: glucose-excited neurons
GI neurons: glucose-inhibited neurons
GHRH: growth hormone-releasing hormone
GLUT: glucose transporter
GnRH: gonadotrophin releasing hormone
HGE neurons: high-glucose excited neurons
HGI neurons: high-glucose inhibited neurons
LH: lateral hypothalamus
KATP channel, potassium ATP-dependent channelMBH: mediobasal hypothalamus
MCH: melanin-concentrating hormone
NPY: neuropeptide Y
PO: preoptic area
POMC: pro-opiomelanocortin
PVN: paraventricular nucleus
ROS: reactive oxygen species
SF1: steroidogenic factor 1
SGLT: sodium-glucose cotransporters
TRPC: transient receptor potential canonical
VMN: ventromedial nucleus.
